# MiR-206 conjugated gold nanoparticle based targeted therapy in breast cancer cells

**DOI:** 10.1038/s41598-022-08185-1

**Published:** 2022-03-18

**Authors:** Ramesh Chaudhari, Simran Nasra, Nikita Meghani, Ashutosh Kumar

**Affiliations:** Biological & Life Sciences, School of Arts & Sciences, Ahmedabad University, Central Campus, Navrangpura, Ahmedabad, Gujarat 380009 India

**Keywords:** Cancer therapy, Nanobiotechnology, Nanomedicine

## Abstract

MicroRNAs (miRNAs) are single-stranded, non-coding, 19–25 nucleotide RNA molecules that have been observed to be dysregulated in many diseases including cancer. miRNAs have been known to play an important role in cellular proliferation, differentiation, migration, apoptosis, survival, and morphogenesis. Breast cancer is heterogeneous in nature and contributed extensively to the increased mortality rate. miRNA can either be tumor-suppressive or oncogenic in nature. The level of expression of miRNA changes according to the subtypes of cancer and the mutation responsible for different cancers. miRNA mimicry or inhibition are emerging possible therapies to maintain the level of miRNA inside the cells. In order to have proper miRNA mimicry, the major hurdle is to deliver the miRNA mimics at the site of tumor. Metallic nanoparticles with modified surface can be used to solve the problem of miRNA delivery. MiR-206 is reported to be down-regulated in Luminal-A type of breast cancer. In the current manuscript, we aim to modify the surface of gold-nanoparticles (AuNPs) with PEG moiety and allow miRNA to attach to it. The fabricated nano-complex, not only delivered miR-206 but also caused cell death in MCF-7 by arresting cells in the G0-G1 phase and inducing apoptosis by downregulating NOTCH 3.

## Introduction

Breast cancer is one of the most common, amongst all the female malignancies in the world and is known to cause development of chemo-resistance and metastatic progression of cancerous tissue^1,2^. Breast cancer is a highly heterogeneous disease, containing distinct yet complex histopathological patterns and clinical behaviours. In clinical facilities across, these intrinsic subtypes of cancer are recognized in the presence of immune-histochemical molecules, like estrogen receptor (ER), progesterone receptor (PR), HER2 expression and Ki67 labelled index^1,3^. The cancers are classified on the basis of the different immune histochemical molecules present which are also due to the difference in subsets of genetic and epigenetic abnormalities present in them^4^. Approximately 70% of breast cancers are ER-positive and/or PR-positive, hence the focus of the present study is on Luminal A, a type of breast cancer where the proliferating cells are hormone-receptor positive (estrogen-receptor and/or progesterone-receptor positive), HER2 negative, and has low levels of the protein Ki-67, which subsequently facilitates in controlling the growth of cells^5^. Previous studies have demonstrated differential expression of miRNA in different types of breast cells, including in different intrinsic subtype (as differentiated by the expression of different receptors) of breast cancer^6,7^.

MiRNAs are known to be small, non-coding RNA molecule which regulates the translation and stability of mRNAs at the post-transcriptional level^8–10^. In mammals, miRNA binding sites are found in 3′ untranslated regions (3′ UTRs), and its binding to the 3′ UTR halts translation or induces mRNA degradation, resulting in gene silencing^10^. It is now well established that dysregulation of these miRNAs can influence the expression of oncogenes or pacify the expression of tumor suppressor genes. The dysregulation of certain microRNAs, which are known as oncomiR has been linked with specific cancer giving rise to particular oncogenic events. MiRNAs are either over-expressed (oncomiR) or down-regulated (anti-oncomiR) in cells, both of which can lead to cancer. Thus, it is necessary to maintain the level of miRNA throughout the cells either via silencing them or by delivering the mimics of miRNA. However, the major concern is the lack of an efficient and proper delivery system for miRNA.

Transfection reagents such as cationic lipids or polymers are used world-wide to deliver the miRNA, but their own cytotoxicity and low transfection efficiency are a big concern^11,12^. To overcome these shortcomings, nanocarriers including gold nanoparticles have been developed and are being tested for delivery of miRNA mimics. The importance of miRNAs in cancer treatment has been recognized increasingly for developing miRNA-based therapies. However, the controlled delivery of miRNAs at target cells in a desired amount and conformation is still a problem^1^. Gold nanoparticles are generally used to deliver miRNA mimics and are hindered by the need of complicated step to conjugate miRNA onto the gold nanoparticle. In this study, we developed a simpler method for the fabrication of miRNA loaded gold nanoparticles to deliver miR-206. This process also has a potential for establishing PEGylated AuNP as the universal carrier for miRNA mimic.

MiR-29, miR-10a, miR-99a, miR-103, miR-146, and miR-206 have been reported to be associated with Luminal-A type of Breast Cancer. MiR-206 functions as a tumor suppressor, inhibiting cell growth, migration and invasion and on down regulation causes apoptosis^11^. MiR-206 levels in breast cancer cells when compared to normal breast cells are dramatically down regulated^12,13^. When up-regulated in breast cancer, miR-206 was able to suppress breast cancer cell proliferation and colony formation by blocking the G1/S transition by targeting cyclin D2^11,14^. Inhibition of the growth of rhabdomyosarcoma (RMS), breast cancer, endometrial endometrioid carcinoma (EEC), lung cancer, and HeLa cells has been shown by the ectopic expression of miR-206^15,16^. Cell invasive and migratory ability has also been shown to be impaired by miR-206 in RMS, EEC, lung cancer, and HeLa cells^15^. Hence, miR-206 has been used in the study as it is known to be down regulated in Luminal A type of breast cancer^17^.

In the present study, we employed a functionalized AuNPs system for the delivery of miR-206 mimic and demonstrated that miRNA when delivered through gold nanoparticles is effective in treating Luminal A type of breast cancer by targeting the NOTCH 3 gene. Consequently, this nano-conjugate has the potential to be used as a universal delivery system for miRNA at the desired targeted site.

## Results and discussion

### Synthesis of PEG capped AuNPs

**Figure 1:**
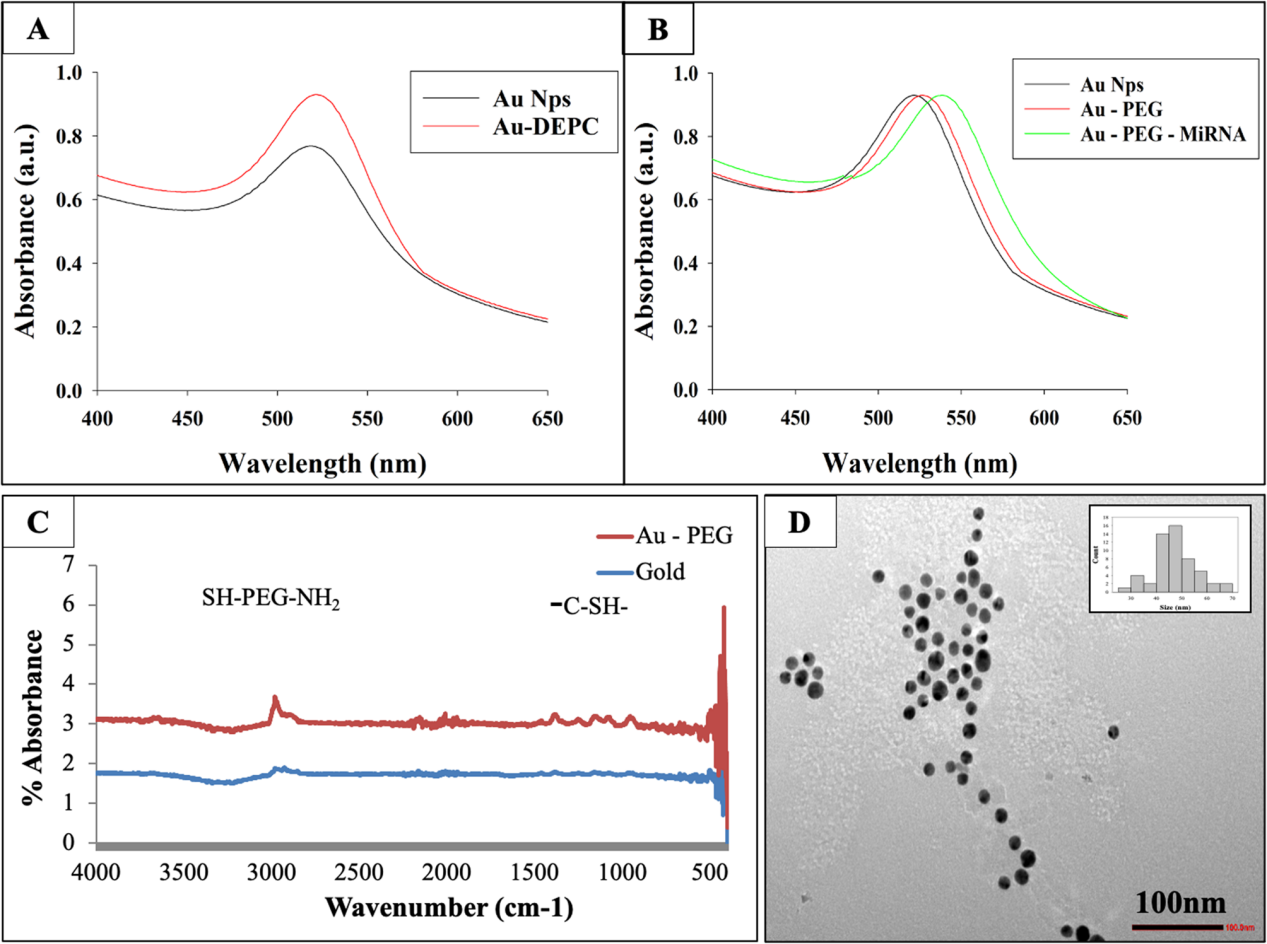
Characterization of formulated gold nanoparticles. (**A**) UV–Vis Spectra obtained for gold nanoparticles and DEPC treated gold nanoparticles. (**B**) UV–Vis Spectra of DEPC treated gold nanoparticles (Au NPs), PEG capped Au NPs (Au—PEG) and MiRNA coated PEG capped Au NPs (Au–PEG–MiRNA). (**C**) FTIR spectra for citrate capped AuNPs (bottom) and MiRNA–PEG (top) capped Au NPs represent the binding of NH_2_-PEG-SH molecule on the surface of AuNPs. (**D**) Transmission Electron Microscopy (TEM) image obtained for PEG coated gold nanoparticles inset: histogram indicating particle size.

Gold nanoparticles (AuNPs) have received attention as a non-viral gene delivery vector due to their unique physicochemical properties such as shape, surface area, amphiphilicity, biocompatibility, and better gene transfection efficiency. Researchers have developed amine-functionalized AuNPs for efficient intracellular delivery, however, the approach was limited to delivering chemically modified siRNAs only^18^. It is highly likely that the function of miRNAs may be impacted by these modifications. Large-scale production, annealing sense and antisense strands to make duplexes, and base pairing with target messenger RNAs (mRNAs) have been largely impeded by these modifications. In addition, these non-viral nano-vectors are limited by drawbacks like low encapsulation efficiency, poor storage stability and slow endosomal escape^19^. AuNPs can be potentially toxic sometimes because of the use of a reducing agent in its synthesis such as NaBH_4_, cysteamine, CTAB, and others^19^. Hence, in order to limit their toxicity, the citrate capping method was used. All the experiments were conducted using DEPC treated AuNPs, with the purpose of escaping any RNAases that can potentially degrade the miRNA. The effect of these treatments on the properties of AuNPs was assessed using UV spectroscopy and transmission electron microscopy (TEM) and our observation suggest no change in the morphology of AuNPs (Fig. 1). A robust loading of miRNA on AuNPs is a two-step process, the first of which is to attach NH_2_-PEG-SH, which will not only make AuNPs, stable and less toxic but also will impart a positive zeta potential which is a cue for miRNA to bind. NH_2_-PEG-SH capped AuNPs were further incubated with miRNA206 mimic (miR-206) for its attachment and the excess NH_2_-PEG-SH was removed.

### Characterization of miR-206 mimic loaded AuNPs

The UV–Vis spectral analysis of AuNPs and DEPC treated AuNPs exhibited a change of 1 nm in wavelength with a slight increase in intensity as observed in the spectrum of DEPC treated AuNPs (Fig. 1A). This could be attributed to the additional temperature given to the AuNPs when autoclaved. It has been reported that DEPC treated AuNPs and AuNPs do not have any difference in their physicochemical properties^20^. The oscillating electric fields of a light ray spreading near a colloidal nanoparticle interrelate with the free electrons causing a combined oscillation of electron charge that is in resonance with the frequency of visible light. These resonant oscillations are termed as surface plasmons. For 30 nm, monodisperse gold nanoparticles, the surface plasmon resonance phenomenon causes absorption of light in the blue-green area of the spectrum (~ 450 nm) while red light (~ 700 nm) is reflected, generating a rich red color.

DEPC treated AuNPs were allowed to bind first with NH_2_-PEG-SH and then with miRNA. The UV–Vis spectral analysis of AuNPs, PEG – AuNPs, and miRNA—PEG—AuNPs showed a shift in the plasmonic resonance peak position of 6 nm between AuNPs and PEG – AuNPs and 10 nm between PEG – AuNPs and miRNA—PEG—AuNPs (Fig. 1B). This shift could be attributed to an increase in the size of the nanoparticles possibly due to the binding of NH_2_-PEG-SH molecule on the surface of AuNPs and then subsequent attachment of miRNA. The binding of NH_2_-PEG-SH molecule on the surface of AuNPs has also been confirmed by the difference in peaks observed in FTIR spectra of both the molecules. The presence of a peak at wavelength 2999/cm and 1237/cm represents the presence of thiol and amine group respectively (Fig. 1C). It has also been reported that bands near 600, 794, 1306, and 1631/cm are due to miRNA, while in the present study, the absorbance was observed at 509, 803, and 1399/cm possibly due to the association of miRNA with NH_2_-PEG-SH capped AuNPs^21^.

The increase in the hydrodynamic size from 20.10 to 48.89 nm also confirms the attachment of PEG molecules on AuNPs. Additionally, the binding of PEG on Au nanoparticles lead to a shift in zeta potential from − 29.3 to + 12.5 mV, as NH_2_-PEG-SH is known to impart a positive charge. These positively charged PEG-AuNPs are then allowed to bind with negatively charged miR-206 mimic, which further results in an increased hydrodynamic size of around 16 nm and negative zeta potential of 16.4 mV (Table 1). It has also been observed that the positively charged AuNPs can form polymeric complexes with the negatively charged therapeutic agents such as miRNA, siRNA, etc. via electrostatic interactions that take place between the phosphate group of miRNA and amine group on the surface of PEG-AuNPs^22^.

**Table 1:** Hydrodynamic size and zeta potential of different nano-formulation using dynamic light scattering.

	Hydrodynamic Size (d nm)	PDI	Zeta potential (mV)
Gold nanoparticles (AuNPs)	19.96 ± 0.03	0.266 ± 0.007	− 26.6 ± 1.35
DEPC treated AuNPs	20.10 ± 0.01	0.260 ± 0.011	− 29.3 ± 1.71
AuNPs–PEG	48.89 ± 0.13	0.370 ± 0.008	12.5 ± 2.71
AuNPs–PEG–miR negative control	64.94 ± 0.12	0.559 ± 0.005	− 16.4 ± 1.15
AuNPs–PEG–miR 206	65.27 ± 0.34	0.602 ± 0.006	− 18.8 ± 1.04

### Loading efficiency of miRNA on to the PEG—AuNPs

**Figure 2:**
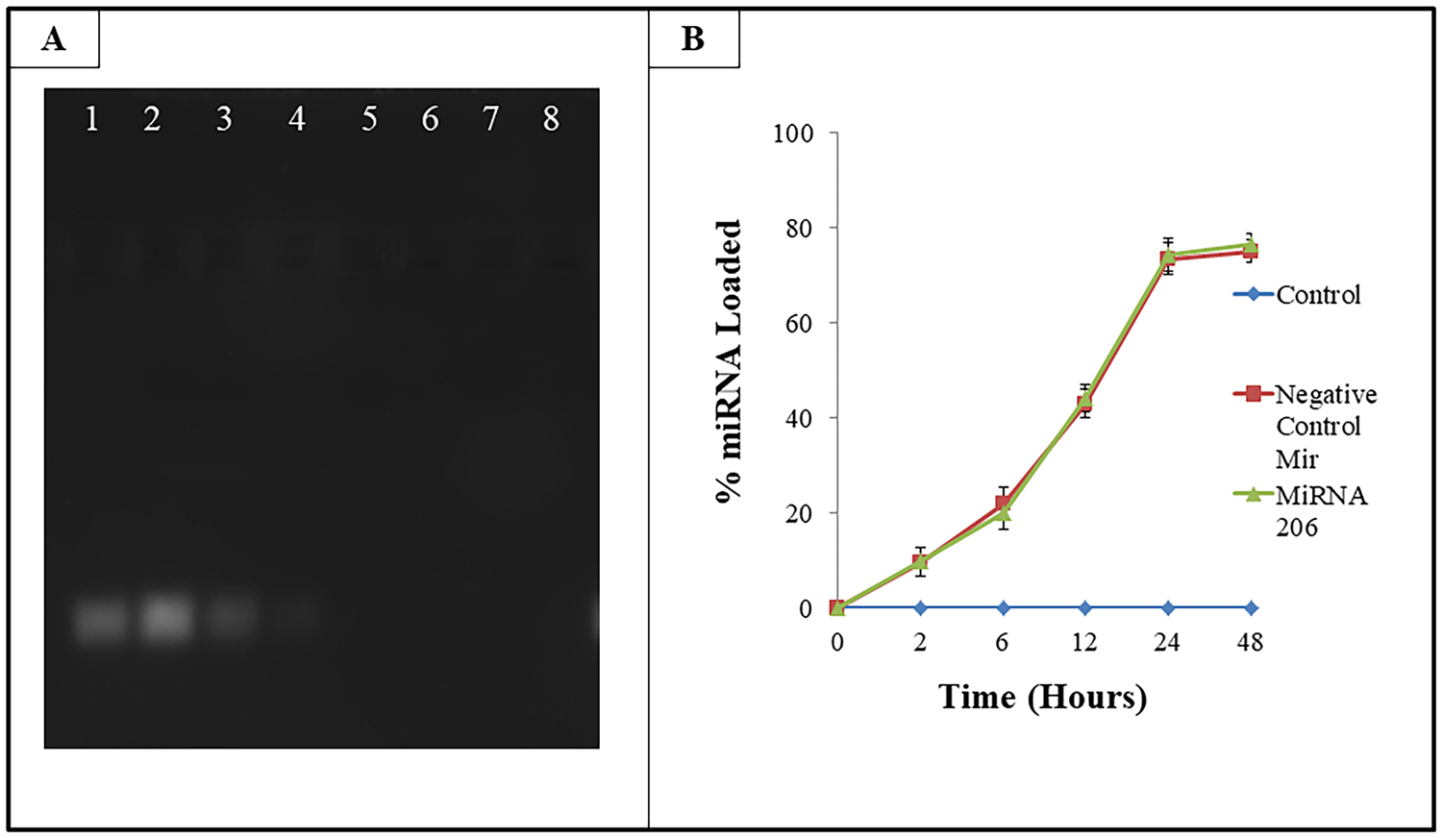
Percent loading efficiency of gold nanoparticles (**A**) Agarose gel electrophoresis of PEG Capped AuNPs incubated with miRNA at various time points. (1) 50 nM miRNA (2)100 nM miRNA (3) AuNPS + miRNA (2 h) (4) AuNPS + miRNA (4 h) (5) AuNPs + miRNA (6 h) (6) AuNPS + miRNA (12 h) (7) AuNPs + miRNA (24 h) (8) AuNPs + miRNA (48 h) (full-length gel image has been included in a “Supplementary Information” file) (**B**) Line plot showing percent loading of miRNA on PEG capped AuNPs. The data represents mean ± S.E. of three independent experiments.

PEG capped AuNPs were incubated and stirred with 100 nM of miRNA for different time points. After the incubation, the nanoparticles were centrifuged and unbound miRNA in the supernatant was measured using a spectrophotometer. The negative control miR is a random sequence miRNA mimic molecule that has been extensively tested in human cell lines and tissues and validated to not produce identifiable effects on known miRNA function. The binding of miRNA to PEG-AuNPs was also assessed using agarose get electrophoresis. The band visibility in (Fig. 2A) indicates the presence of unbound miRNA in lanes 1, 2, 3, and 4, while the lack of visibility of miRNA in lanes 5, 6, 7 and 8 provides the support for the complete binding of miRNA to PEG -AuNPs. The binding of miRNA was also confirmed by plotting percent miRNA binding over time (Fig. 2B). A time dependent increase in the binding efficiency of the miRNA was also observed at 0, 2, 6, 12 h. However, a plateau was observed after 24 h, with a binding efficiency of 74% and 76% at 24 and 48 h respectively. Hence, for a further experiment, the miRNA was allowed to bind only for 24 h.

### Cytotoxicity of miRNA—PEG—AuNPs

**Figure 3:**
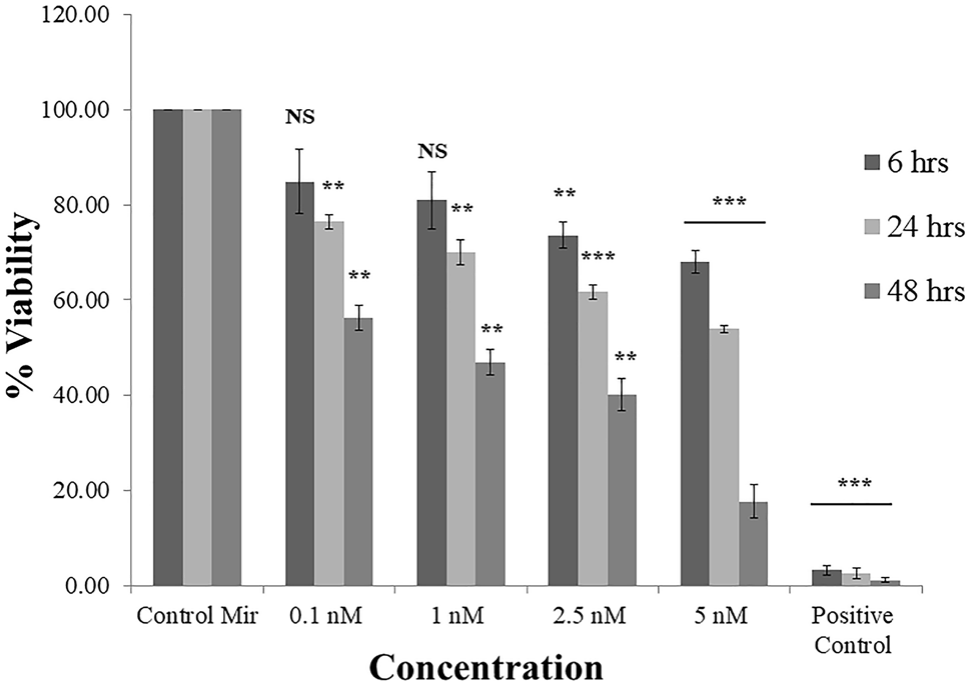
Concentration and time dependent percent MTT reduction of miRNA coated Au Nps in MCF-7 cells. A negative control miRNA coated with Au Nps was considered as Control miRNA. The viability of the control cells was considered as 100%. Values represents mean ± S.E. of three independent experiments (*p < 0.05, **p < 0.001).

Cytotoxicity of the formulation was assessed using cell viability assay also known as MTT assay. MiR-206 has been reported to be down regulated in Luminal A type of breast cancer, hence MCF-7 cells were selected for assessing the effect of miRNA–PEG–AuNPs. Negative control miRNA treatment was also given to the cells and the observed response was considered as control. A substantial decrease in the cells number was observed when exposed to different concentrations of miRNA. All the concentrations showed high toxicity after 48 h of treatment. Ethyl methanesulfonate (EMS) was used as a positive control (Fig. 3).

At treatment concentration 5 nM, the cell viability drops to 68.10% and 53.88% for 6 and 24 h respectively. However, it has dropped to 17.60% after 48 h of exposure. This data indicates that when downregulated, miR-206 was transfected with gold nanocomplex resulting in a higher cellular level of miR-206 and it helps in reducing cell viability. This also provides evidence for the fact that the PEG-AuNPs is not only an efficient method of delivery but also can safely deliver sensitive therapeutic agents with desired concentrations for effective treatment. It has been reported that when down-regulated miRNA are introduced into cells it facilitates apoptosis resulting in cell death^23^.

### Cell cycle progression analysis

**Figure 4:**
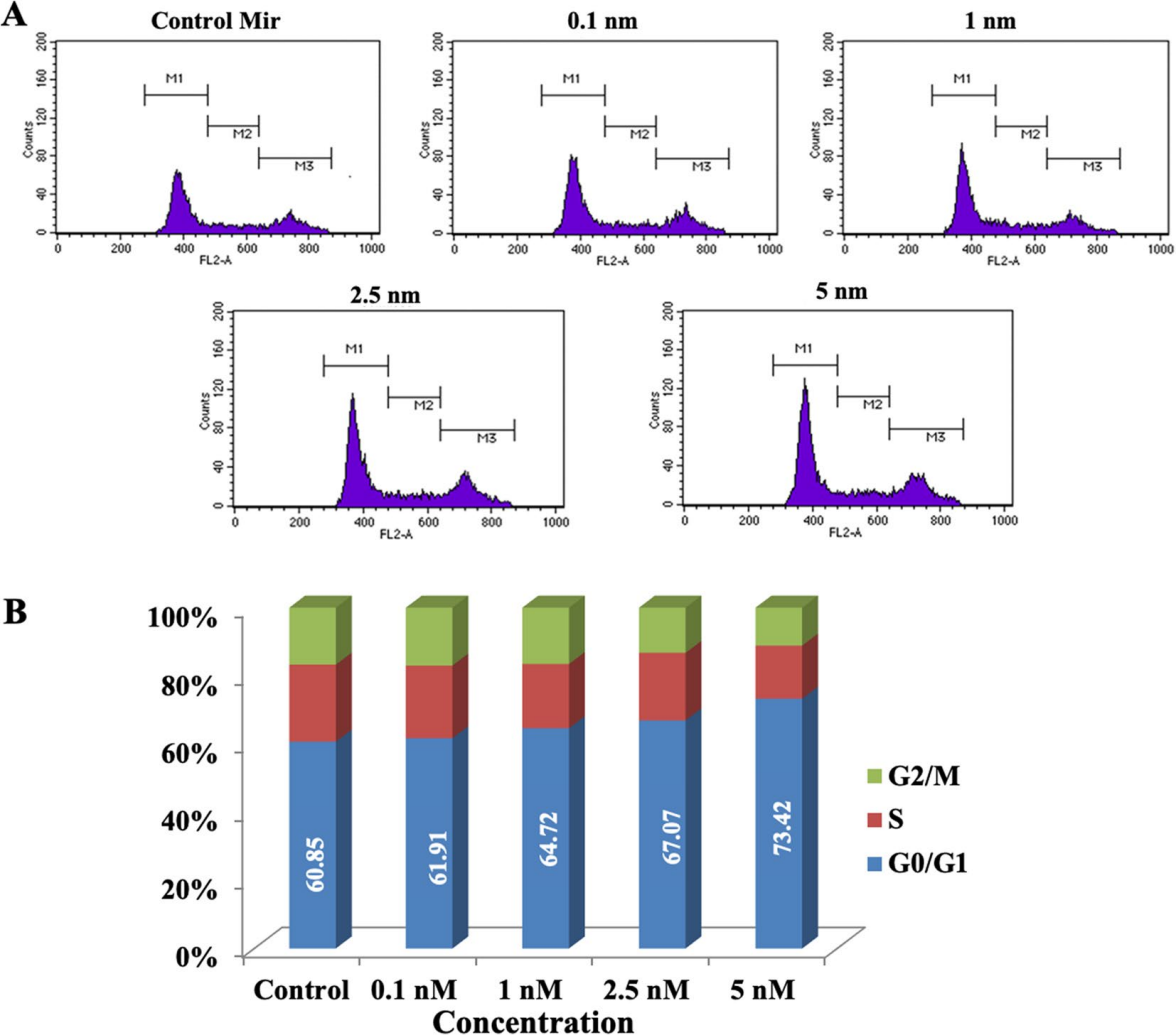
The cell cycle distribution analysis using flow cytometry in MCF-7 cells. (**A**) The cell cycle distribution histogram of cells treated with 0.1 nM, 1 nM, 2.5 nM and 5 nM of miRNA -206 mimic coated Au Nps for 24 h. (**B**) Graphical representation of cell cycle arrest after 24 h.

Cell cycle progression was analysed using propidium iodide (PI) uptake assay in a flow cytometer. MiR-206 at all concentration was able to arrest the MCF-7 cells in G0/G1 phase. The arrest in G0/G1 phase increased in a concentration dependent manner with the maximum arrest observed at concentration 5 nM after exposure of 24 h (Fig. 4). In addition, a concentration dependent decrease in the population of G2-M and S phase across all concentration was observed. As miR-206 arrests cells in G1 phase and regulates cell proliferation, hence its down regulation plays a vital role in cancer progression^24^.

### Apoptosis marker

#### Mitochondrial membrane potential

**Figure 5:**
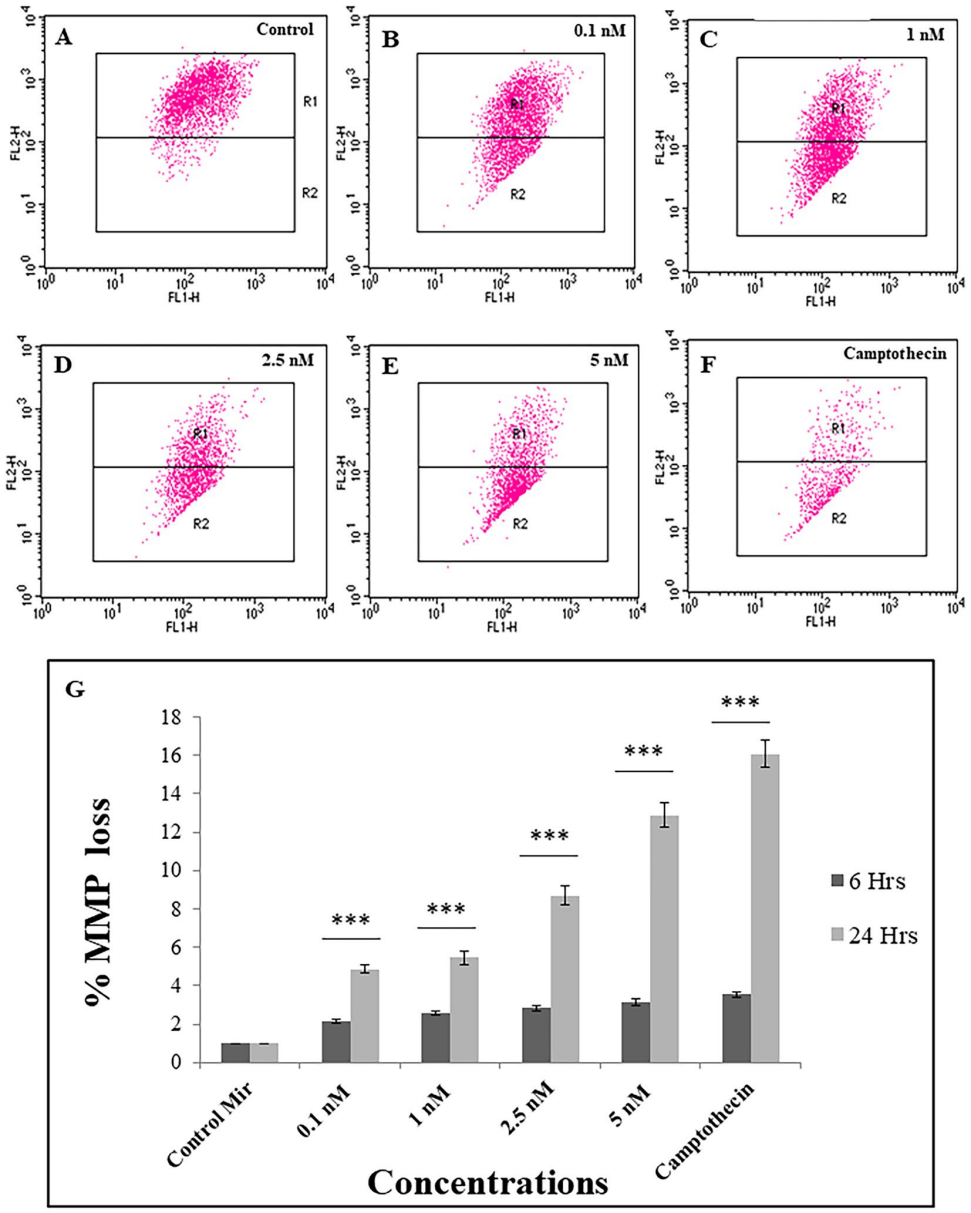
Evaluation of mitochondrial membrane potential (MMP) in MCF-7 cells using flow cytometer. (**A**–**E**) Flow cytometric scatter plot for JC-1 in cells treated with different concentration of miRNA coated AuNPs (**F**) camptothecin (**G**) Bar graph represents the percentage of JC-1 monomer positive cells (%MMP loss). Values represents mean ± S.E. of three independent experiments (*p < 0.05, **p < 0.001).

JC-1 dye was used to determine the mitochondrial membrane potential in a flow cytometer. MCF-7 cells were treated with control miRNA and miRNA coated on PEGylated AuNPs for 24 h and were assessed for alteration in their mitochondrial membrane potential (MMP). A significant (p < 0.05) increase in green fluorescence intensity 4.87–16.05% was observed at concentration 0.1–5 nM at 24 h (Fig. 5). However, no significant increase in green fluorescence was observed across concentrations after 6 h of exposure. The increase in green fluorescence suggests the involvement of the mitochondria in the activation of apoptotic pathways. As the electron transport system of the cell is located at mitochondria and any disturbance in the MMP can lead to altered respiration and overall cellular metabolism.

#### Annexin V binding assay

**Figure 6:**
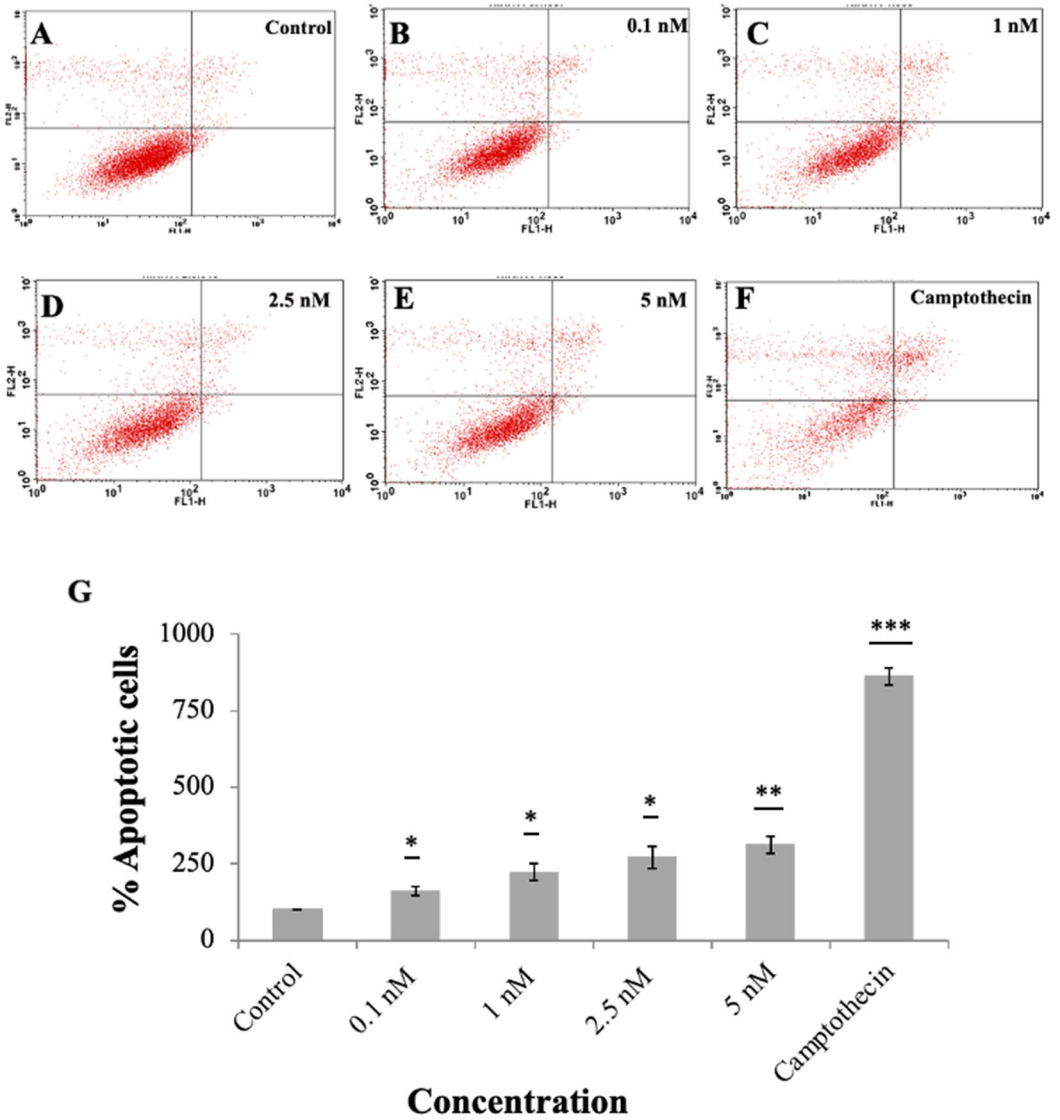
Estimation of apoptosis in MCF-7 cells after treatment. Flow cytometric scatter plot of PI vs Annexin-FITC (**A**–**E**) various concentration of miRNA coated AuNPs (**F**) Camptothecin (1 µg/mL) was used as positive control. (**G**) Bar graph represents the percent apoptotic population induced by miRNA coated Au Nps. Values represents mean ± S.E. of three independent experiments (*p < 0.05, **p < 0.001).

Annexin V-PI dual staining assay was performed to assess the apoptotic cells. A significant increase in the percent apoptotic cells was observed across all concentrations with an increase in 60% apoptotic cells at concentration 0.1 nM after 24 h (Fig. 6). The data exhibits the presence of early apoptotic cells after the exposure with both AuNPs. This observation is also concurrent with the observations made in the loss of MMP at 24 h. This is also consistent with the previous studies that showed the occurrence of apoptosis after cells were treated with 206 miRNA^17^.

### Cellular pathway analysis

**Figure 7:**
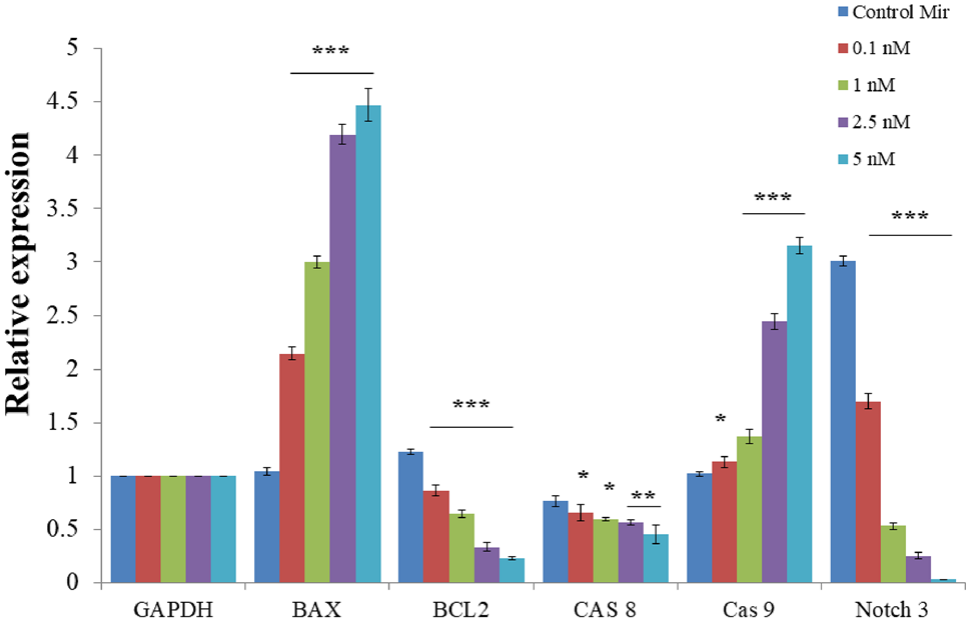
Determining Relative Expression of genes in MCF-7 cells after exposed to various concentration of MiRNA–PEG–Au NPs for 24 h using RT-qPCR. GAPDH was considered as endogenous control while expressions of BAX, BCL2, Caspase 8, 9 and Notch 3 were studied due to increase in mitochondrial membrane potential and NOTCH 3 being directly associated with miRNA 206. Values represents mean ± S.E. of three independent experiments (*p < 0.05, **p < 0.001).

Cellular pathway analysis was conducted by analysing the mRNA expression pattern of target genes using real time PCR. The increase in mitochondrial membrane potential and presence of apoptotic cells provides an indication towards the activation of mitochondria mediated apoptosis. Hence, to explore this pathway in detail the expression of a regulatory gene such as BAX, BCL2, Caspase 8, and 9 were selected. NOTCH 3 is a gene associated with the down regulation of miR-206. Hence MCF-7 cells were treated with various concentrations of miRNA nanocomplex for 24 h. A concentration dependent increase in BAX expression and decrease in BCL2 expression was observed in the treated cells (Fig. 7). This is consistent with the increase in mitochondrial membrane potential thus confirming the mitochondrial activation of apoptosis. However, the lack of significant change in expression of both the caspases suggests that a caspase independent pathway is activated which is also a common phenomenon in breast cancer cells^25,26^. However, in order to confirm that the activation is caspase independent the gene expression of caspase 6 and 7 too need to be studied further.

Various studies acknowledge that miR-206 is a tumor suppressor that is downregulated in various tumors and is involved in cancer metastasis. *NOTCH3*, an established target of miR-206, has also been reported to be often expressed in cancer samples and has a capacity in the modulation of cell proliferation and tumorigenic potential in certain xenograft models^27^. The observation demonstrates a significant decrease in NOTCH 3 expression and BCL2 expression while the simultaneous increase in BAX expression is a route via which miR-206 works, confirming the up-regulation of miR-206 directly links to the down regulation of NOTCH 3.

## Conclusion

In the present manuscript, an efficient method for delivering sensitive therapeutic agent like miRNA has been demonstrated, and a similar approach can be used to deliver siRNA and other therapeutic agents. The synthesized nano-complex contains AuNPs and NH_2_-PEG-SH and together form PEGylated AuNPs. Apart from being able to electrostatically bind to miRNA, the nano-complex has a major advantage of easy to conjugate with therapeutic agents. A small concentration in nanomolar (nM) loaded onto nano-complex is able to cause cell death of cancer cells. MiR-206 when given via gold nano-complex was able to stop cell proliferation, induce G0-G1 cell arrest and change the mitochondrial membrane potential.

**Figure 8:**
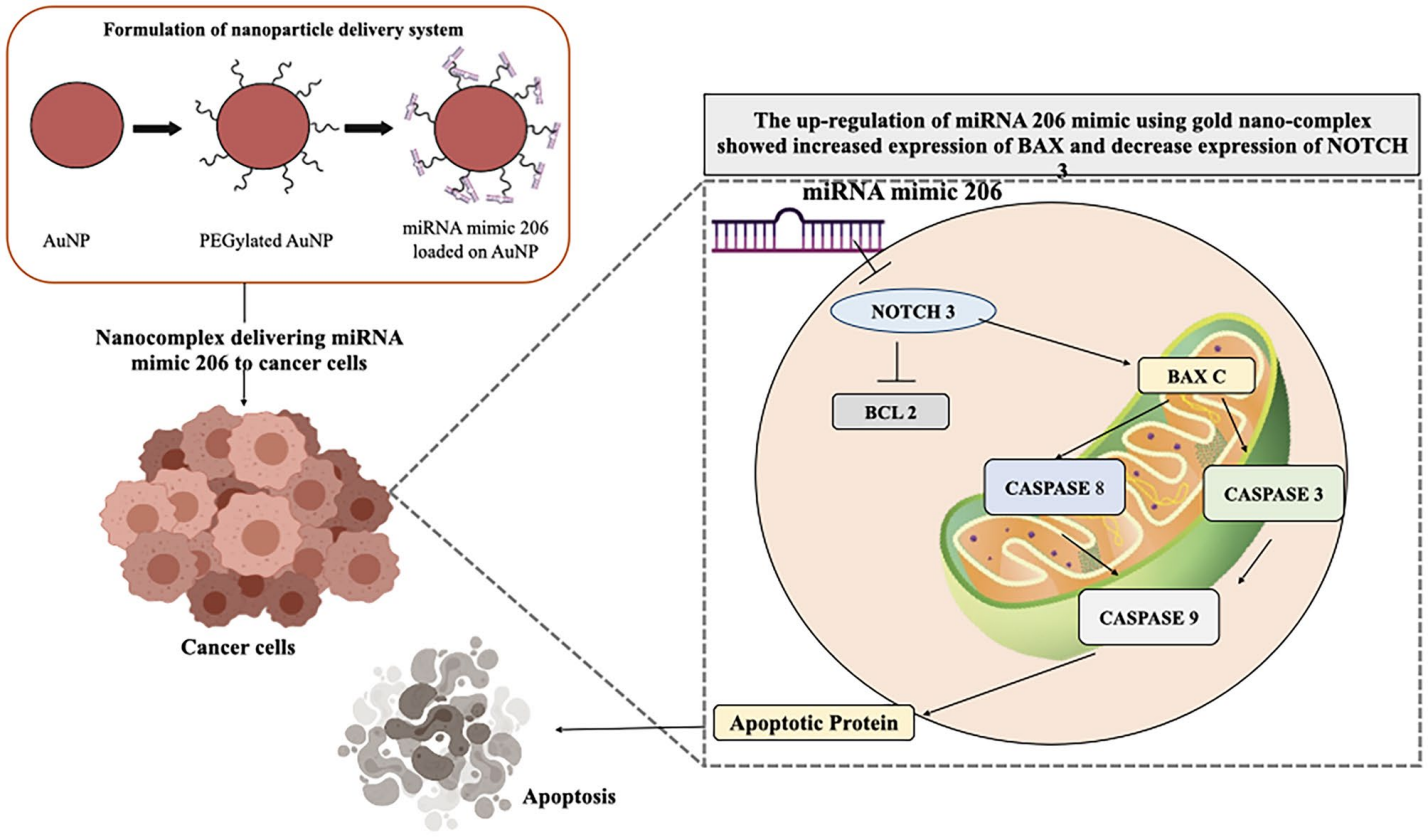
Schematic representation of the study.

MiR-206 works via different pathways in different cells; it is often down-regulated via the up-regulation of NOTCH 3 or in some rare cases up regulated and is associated with the Cyclin B1. However, in almost all different types of breast cancer, miR-206 has been reported to be down regulated and is said to target NOTCH 3. The up regulation of miR-206 mimic using gold nano-complex, not only showed increased expression of BAX but also decrease expression of NOTCH 3, which suggests that the miRNA delivered to the cells in intact conformation and there are no structural or any sort of changes in the miRNA which might lead to loss of activity of miRNA-206 mimic delivered to the cells (Fig. 8). Thus, the described method can be used as a potential alternative to deliver the therapeutic agents.

## Materials and methods

### Materials

Chloroauric acid (HAuCl_4_), sodium citrate (Na_3_C_6_H_5_O_7_), diethylpyrocarbonate (DEPC) were obtained from Invitrogen, Ethyl Methane Sulfonate (EMS), Polyethylene glycol (PEG average molecular weight (M_n_-800)) were procured from HiMedia, miRNA − 206 mimic (UGGAAUGUAAGGAAGUGUGUGG), negative miRNA control (GGUUCGUACGUACACUGUUCA) from Sigma, Agarose, Ethidium bromide (EtBr), 2 M Tris base, acetic acid, 0.5 M EDTA (pH 8), Milli Q water, loading dye, Modified Eagle’s Media (MEM), Fetal Bovine Serum (FBS), sodium bicarbonate (NaHCO_3_), Gibco Antibiotic–Antimycotic solution, 3-(4,5-dimethylthiazol-2-yl)-2,5-diphenyltetrazolium bromide (MTT), Dimethylsulfoxide (DMSO), 5,5,6,6′-tetrachloro-1,1′,3,3′ tetraethylbenzimi-dazoylcarbocyanine iodide (JC-1) dye, FITC-Annexin V, Propidium iodide, TRIzol Reagent, and Triton-X100 were obtained from Thermo Fisher.

### Synthesis of gold nanoparticle

The synthesis of citrate-capped gold nanoparticles (AuNPs) of 13 nm diameter was done using the method reported by Crew et al.^21^. Concisely, 5 mL of freshly prepared 38.8 mM sodium citrate (Na_3_C_6_H_5_O_7_) was added to a boiling solution containing 45 mL of 1 mM chloroauric acid (HAuCl_4_). The solution was heated for 30 min. Subsequently, the synthesized citrate-capped gold nanoparticles were treated with 0.1% diethylpyrocarbonate (DEPC) for 12 h alongside stirring and then autoclaved at 121 °C for 60 min.

### Capping of PEG on synthesized AuNPs

The PEG capped AuNPs (PEG-AuNPs) were synthesized using a method reported by Manson et al.^28^ with slight modification. In order to synthesize PEG capped AuNPs, 16.8 μg of NH_2_-PEG-SH was added to the 1 mL of DEPC treated AuNP solution (1.9322 mM) at room temperature. The solution was agitated at room temperature for 2 h to assist for the exchange of the citrate molecules with PEG molecules. Centrifugation of the obtained AuNPs was performed using Centrifuge 5424 R, Eppendorf at 10,000 rpm for 90 min, at 4 °C, in the sets of 1 mL^29^. 900 µL of the supernatant was drawn out, and the pellet of AuNPs was obtained. 900 µL of DEPC treated water was then added and stirred, to make up the volume up to 1 mL.

### MiRNA-206 mimic loading on PEG capped AuNPs

1 mL of NH_2_-PEG-SH capped AuNPs was incubated with 1000 nM of miRNA206 mimic and stirred at 25 °C in Thermomixer comfort, Eppendorf for 24 h. Subsequently, the solution containing the formulated nanoparticles was centrifuge at 10,000 rpm, for 30 min, at 4 °C to eliminate the unbound miRNA. The nanoparticles were then re-suspended in DEPC treated water. Similarly, the negative control miRNA nanoparticles were synthesized to be used as the negative control.

### Characterization of miR-206 mimic loaded AuNPs

Characterization of the following samples and controls were performed:

A. Citrate capped AuNPs,
B. DEPC treated AuNPs (AuNPs),
C. PEG capped AuNPs (PEG–AuNPs),
D. PEG capped AuNPs loaded with Negative miRNA control (miR-Neg–PEG–AuNPs)
E. PEG capped AuNPs loaded with miR-206 mimic (miR-206–PEG–AuNPs)

UV–visible spectra for each sample were recorded using a SYNERGY-HT multiwell plate reader (Bio-Tek, USA) using the Gen5 software in the range of 300–700 nm, at 1 nm increments in the wavelength.

Hydrodynamic size and zeta potential of the above listed samples were determined by transferring 1% aqueous solution of nanoparticles into a disposable polystyrene cuvette and standard zeta cuvette and measured using a Zetasizer Nano-ZS equipped with 4.0 Mw, 633 nm laser (Model ZEN3600, Malvern Instruments, Malvern, UK). The samples were analysed three times at 25 °C.

Infrared spectra of DEPC treated AuNPs and PEG capped AuNPs were obtained using PerkinElmer FT-IR Spectrometer. The aqueous solution of samples was dried and mixed with potassium bromide (KBr) to obtain a fine powder which was pressed onto the discs. All spectra were measured at a resolution of 1/cm and over a wavelength range of 4000–400/cm.

### Determining the miRNA loading efficiency

In order to evaluate the loading efficiency of miR-206 mimic and the negative control, onto the PEG capped AuNPs, was obtained by determining free miRNA concentration^30^. PEG capped AuNPs were incubated and stirred with 100 nM of miRNA for a duration of 2, 12, 24, and 48 h. After the incubation, the nanoparticles were centrifuged at 10,000 rpm for 30 min at 4 °C to remove all the unbound miRNA. Unbound miRNA in the supernatant was determined by measuring absorbance with of UV–Vis spectrophotometer at 260, 280, and 320 nm. The miRNA loaded was measured as the percentage of miRNA loaded to the total amount of miRNA added initially.

### Validation of miR-206–PEG–AuNPs using agarose gel electrophoresis

Gel electrophoresis was carried out for the validation of miR-206 conjugation with PEG-AuNPs. After the incubation, the supernatant was collected and subjected to 1% agarose gel containing ethidium bromide dye (EtBr) and run in tris acetate buffer at 110 V for approximately 30 min^22^. The gel was photographed using GE ImageQuant LAS 5000.

### Cell culture

The human breast adenocarcinoma cell line, MCF-7 was obtained from National Centre for Cell Sciences, Pune, India, cultured in MEM media and supplemented with 10% FBS (heat inactivated), 0.2% sodium bicarbonate, and 10 mL/L antibiotic and antimycotic solution, at 37 °C under a humidified atmosphere of 5% CO_2_. Cells were treated with different formulations and miR–Neg–PEG–AuNP was used as a negative control in all the experiments.

### Cell viability assay for the formulated AuNPs on MCF-7 cell-line

The cytotoxic effect of AuNPs was determined by performing an MTT assay according to Mosmann et al.^31^ and with a slight modification with Shukla et al.^32^. In brief, the cells (1 × 10^4^ cells/well) in 100 µL of culture medium were seeded in 96-well plates and incubated for 40 h. Cells were then exposed to different concentrations of 0.1 nM (nano Molar), 1 nM, 2.5 nM, and 5 nM of miR-206–PEG–AuNPs for 6 and 24 h. MiRNA’s interference with the assay reagents was also assessed using a cell-free system, where miRNA’s alone were incubated with the assay reagents, including dye and buffers, and the absorption was monitored by spectroscopy. Negative control miRNA was allowed to go through the same process. The results were assessed by measuring the absorbance of the end product at 595 nm wavelength using a SYNERGY-HT multiwell plate reader (Bio-Tek, USA) using the Gen5 software.

### Propidium iodide uptake assay for cell cycle analysis

The distribution of DNA in the cell cycle was studied by using FACSCalibur, BD Bioscience flow cytometer. About 3 × 10^5^ cells/well were seeded in six-well cell culture plates and the treatment of different concentrations^33^ of nanoparticles was given to cells for 24 h. After the removal of treatment, cells were harvested and centrifuged at 135*g* for 10 min at 37 °C. Ice-cold ethanol (70% in 1 × PBS) was used to fix the pellet which was then incubated at − 20 °C for 30 min. Further centrifugation was carried out, followed by resuspension of pellet in lysis buffer (0.2% triton in 1 × PBS). After incubation at 4 °C for 30 min, cells were centrifuged and resuspended in 20 μg/mL RNase prepared in 1 × PBS and incubated at 37 °C for 30 min. Final centrifugation was carried out and cells were resuspended in 1 × PBS and stained with 10 μL propidium iodide (PI) (1 mg/mL) followed by incubation at 4 °C until analysed by flow cytometry.

### Apoptosis assays

#### Mitochondrial membrane potential

In order to explore the effect of miR-206 on mitochondrial membrane potential, JC-1 dye was given to cells after being treated with miRNA loaded AuNPs following the previously reported studies^34^. Concisely, in a 6 well plate, 3 × 10^5^ cells/well were seeded and treated with AuNPs loaded with miRNA for 6 h and 24 h at concentrations 0.1 nM, 1 nM, 2.5 nM, and 5 nM. 1 μM Camptothecin was used as the positive control. After removal of treatment, cells were washed with PBS and incubated with 10 μM of JC-1 dye for 15 min at 37 °C. The cells were analyzed using a BD FACSCalibur flow cytometer.

#### Annexin V assay

Cells actively undergoing apoptosis were identified by staining with FITC-Annexin V and PI according to the manufacturer's protocol (BD Biosciences, San Jose, CA, USA). Briefly, 3 × 10^5^ cells/well were seeded in a 6 well culture plate for 40 h prior to treatment. Cells were treated with the nanoparticle formulation at different concentrations for 6 and 24 h. 1 μM camptothecin was used as the positive control for this assay. After removal of treatment, cells were harvested and washed with PBS, resuspended in 100 µL binding buffer containing 5 μL of FITC-Annexin V and PI and kept at room temperature in the dark, after incubation, 400 μL of binding buffer was added to each sample and analyzed using flow cytometer.

### RNA isolation and differential gene expression analysis using RT PCR

The total RNA was extracted from cells after delivery of miRNA loaded AuNP using Trizol^35^ at different concentrations. The cells were treated for 6 and 24 h. RNA concentration and quality were determined with a SYNERGY-HT multiwell plate reader (Bio-Tek, USA). cDNA was synthesized using the verso c-DNA synthesis kit. Equal loading was ensured by an internal control (GAPDH). Quantitative real-time PCR was performed with a SYBR Green Kit using the housekeeping gene GAPDH as a control (Table 2). Gene expression was quantified by the delta delta CT method.

**Table 2:** Details of primers used in RT qPCR to assess the gene expression.

Primer name	Primer sequence
GAPDH-forward	CAGGAGGCATTGCTGATGAT
GAPDH-reverse	GAAGGCTGGGGCTCATTT
BAX-forward	CCCGAGAGGTCTTTTTCCGAG
BAX-reverse	CCAGCCCATGATGGTTCTGAT
BCL2-forward	CTGCACCTGACGCCCTTCACC
BCL2-reverse	CACATGACCCCACCGAACTCAAAGA
Caspase 8-forward	GGTCACTTGAACCTTGGGAA
Caspase 8-reverse	AGGCCAGATCTTCACTGTCC
Caspase 9-forward	GTGGACATTGGTTCTGGAGGAT
Caspase 9-reverse	CGCAACTTCTCACAGTCGATG
NOTCH3 forward primer	TCTTGCTGCTGGTCATTCTC
NOTCH3 reverse primer	TGCCTCATCCTCTTCAGTTG

### Statistical analysis

All the experiments were carried out in triplicates and the results were presented as means ± standard error means (SEM). ANOVA was used for statistical analysis followed by Dunnett’s post hoc multiple comparison tests from Graph Prism-8.0. In all the cases, the p-value less than 0.05 was considered as statistically significant.

## Supplementary Information

Supplementary Information.
