# Supplementary material for: MiR-206 conjugated gold nanoparticle based targeted therapy in breast cancer cells
Source: Sci Rep. 2022 Mar 18;12:4713. doi: 10.1038/s41598-022-08185-1 (PMC8933417; doi:10.1038/s41598-022-08185-1)

**
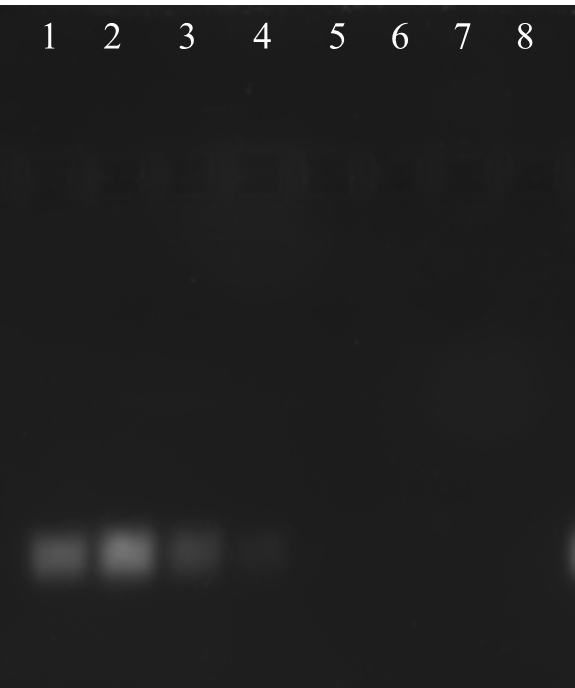
**

**Supplementary fig. 1**

Loading efficiency of gold nanoparticles on Agarose gel electrophoresis of PEG Capped AuNPs incubated with miRNA at various time points. 1) 50nM miRNA 2)100nM miRNA 3) AuNPS + miRNA (2 hours) 4) AuNPS + miRNA (4 hours) 5) AuNPs + miRNA (6 hours) 6) AuNPS + miRNA (12 hours) 7) AuNPs + miRNA (24 hours) 8) AuNPs + miRNA (48 hours)

Exposure_1


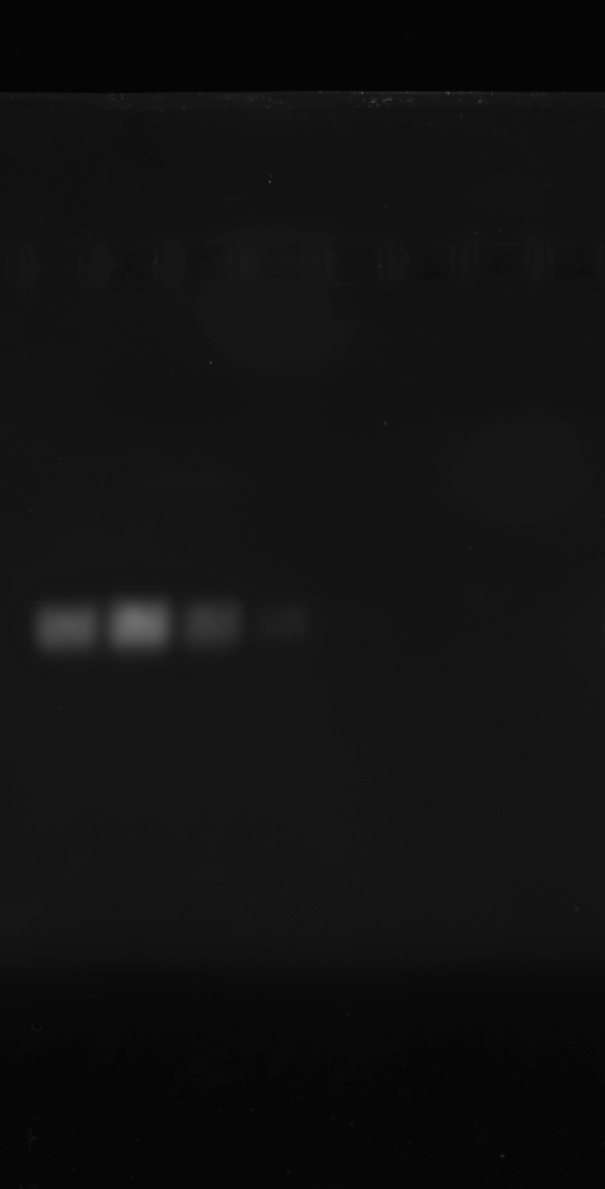


Exposure_2


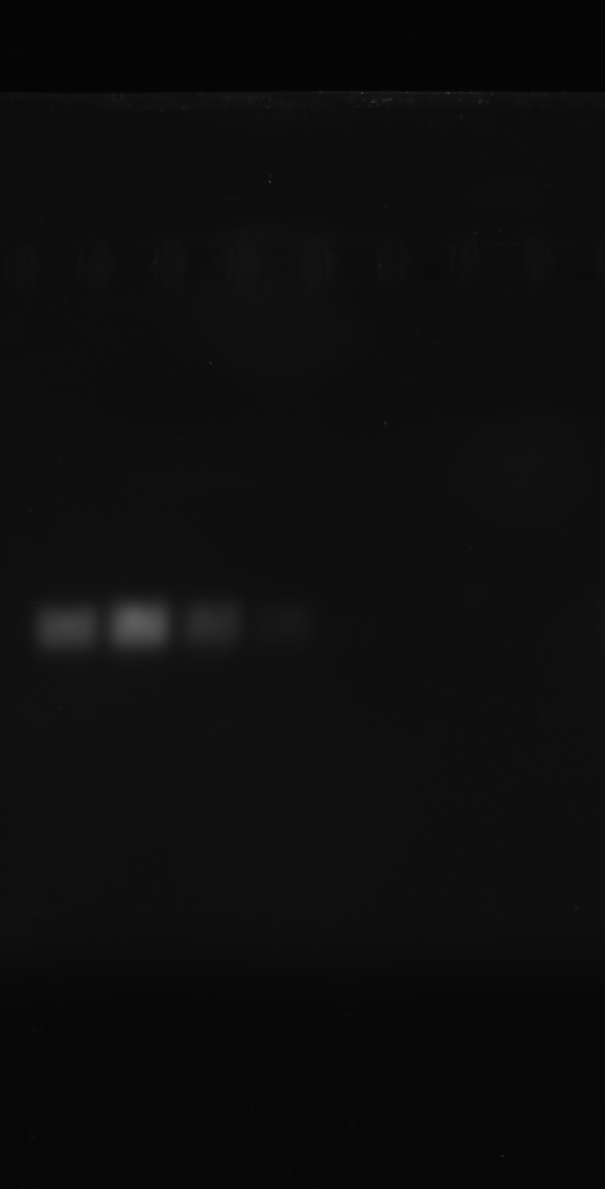


Exposure_3


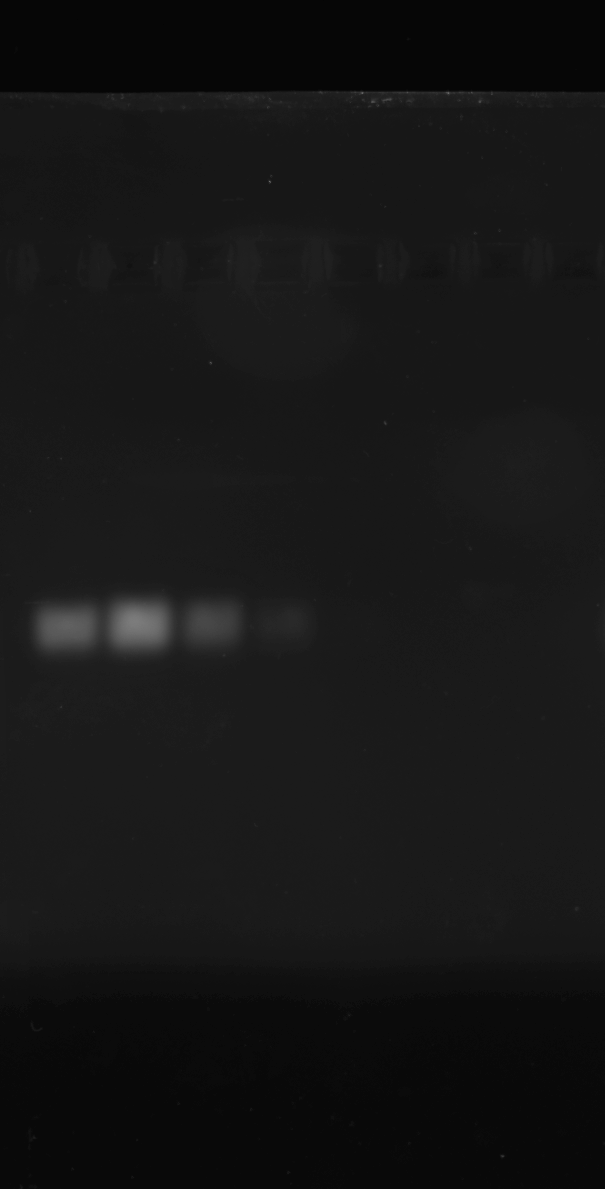


Exposure 4


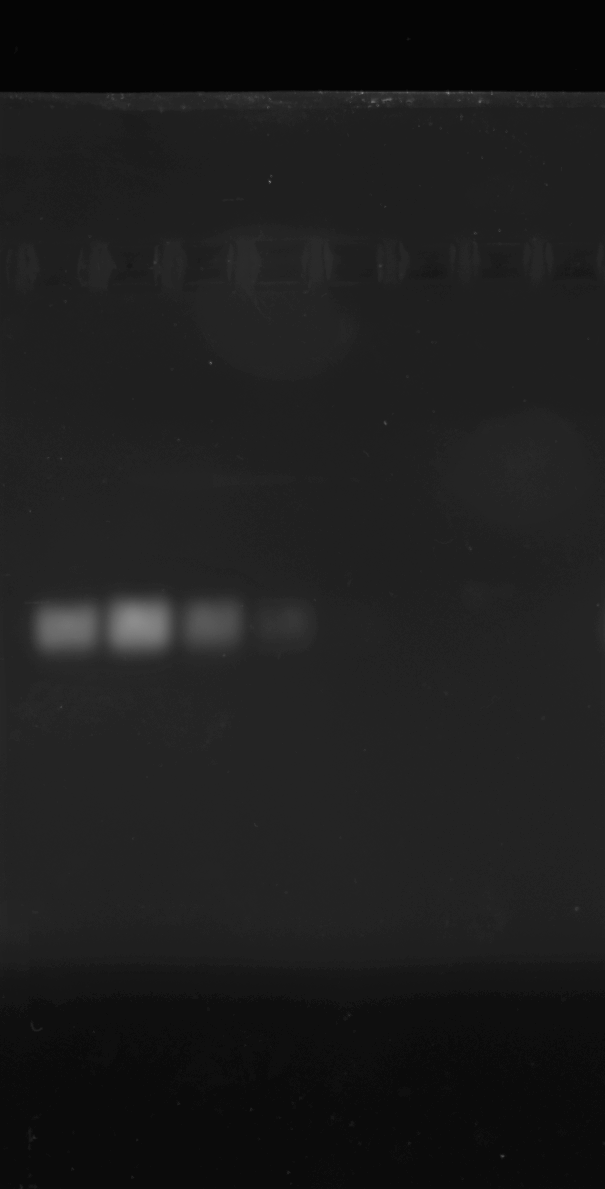


Exposure 5


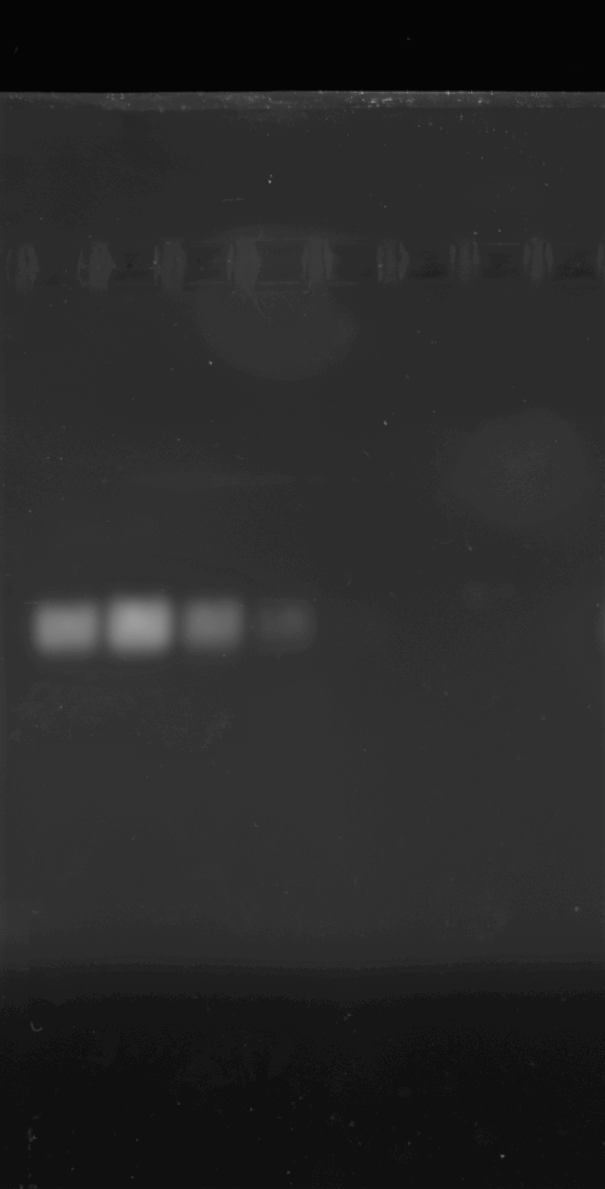


Exposure 6


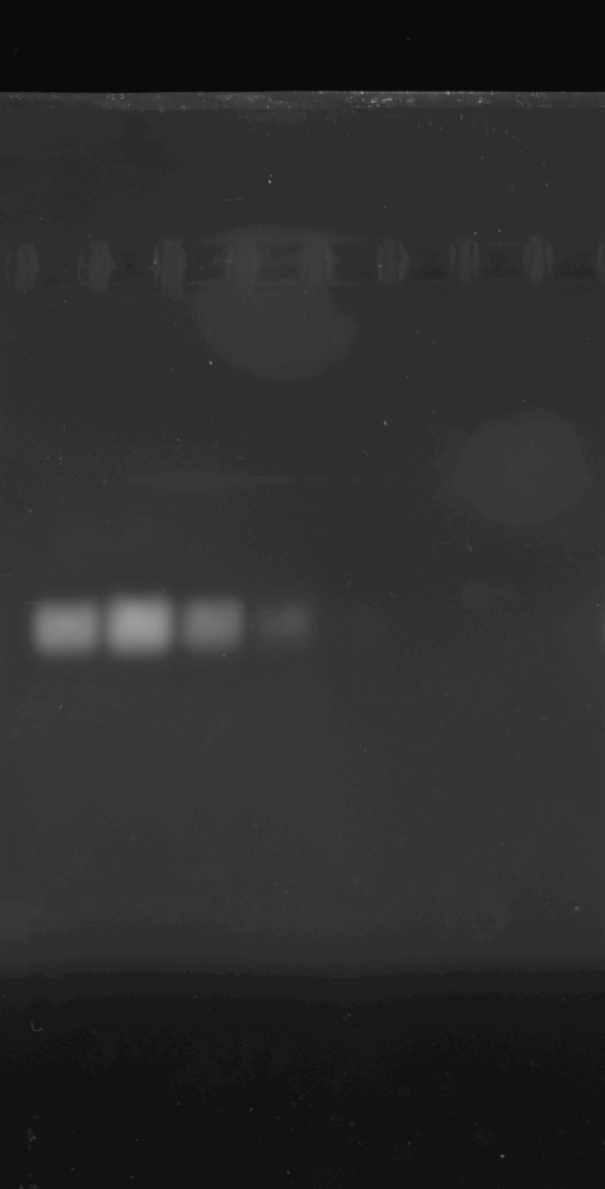

Supplement: Supplementary file 1 — Supplementary Information. [file 41598_2022_8185_MOESM1_ESM.docx]
